# Characterization of 19 polymorphic SSR markers in *Spirodela polyrhiza* (Lemnaceae) and cross‐amplification in *Lemna perpusilla*

**DOI:** 10.1002/aps3.1153

**Published:** 2018-05-31

**Authors:** Nana Xu, Fanglu Hu, Jiameng Wu, Wenjun Zhang, Mengwei Wang, Mingdong Zhu, Jianwei Ke

**Affiliations:** ^1^ Fishery College of Zhejiang Ocean University 1 Haida South Road Zhoushan Zhejiang 316022 People’s Republic of China; ^2^ Ocean Research Center of Zhoushan Zhejiang University 10 Tiyu Road Zhoushan Zhejiang 316021 People’s Republic of China; ^3^ Zhoushan Water Conservancy Survey and Design Institute Zhoushan Zhejiang 316021 People’s Republic of China

**Keywords:** duckweed, *Lemna perpusilla*, Lemnaceae, microsatellites, polymorphic, *Spirodela polyrhiza*

## Abstract

**Premise of the Study:**

Polymorphic microsatellite primers were developed for greater duckweed, *Spirodela polyrhiza* (Lemnaceae), to investigate genetic diversity and structure for application in a bioremediation program.

**Methods and Results:**

A total of 401 publicly available *S*. *polyrhiza* whole‐genome shotgun sequences were searched for simple sequence repeat loci of two or more nucleotides. Of these, 60 primer pairs were selected to analyze 68 individuals of *S. polyrhiza* from three populations. Nineteen polymorphic microsatellite loci were developed. A total of 108 alleles were detected with an average of 5.7 alleles per locus. The levels of expected and observed heterozygosity were 0.0511–0.8669 and 0–0.8750, respectively. Ten loci also successfully amplified in 16 individuals of *Lemna perpusilla*.

**Conclusions:**

The results demonstrate the broad utility of these microsatellite loci for studying population genetics in *S. polyrhiza*.

*Spirodela polyrhiza* (L.) Schleid. (Lemnaceae), commonly known as greater duckweed, has thalloid shoots lacking a stem and, in more derived species, lacking roots. As one of the smallest, fastest‐growing flowering plants, *S*. *polyrhiza* is gaining attention for use in bioremediation of polluted water (Appenroth et al., 2015). Nevertheless, there is significant physiological variation among different individuals of the same species of duckweed. It has been documented that growth rates of most species of duckweed (Ziegler et al., 2015), as well as the formation capacity of turions (i.e., dormant vegetative buds) of *S*. *polyrhiza* (Kuehdorf et al., 2014), depend to a large degree on the individual. Hence, characterization and identification of duckweed individuals are required. In this study, we utilized public genome databases to develop and characterize microsatellite markers for *S. polyrhiza* that can be used for analysis of genetic variation and for future bioremediation applications.

## METHODS AND RESULTS

A total of 68 individuals of *S. polyrhiza* (28 individuals from Zhoushan Island, 24 individuals from Wenzhou, and 16 individuals from Hangzhou), as well as 16 individuals of another common duckweed species (*Lemna perpusilla* Torr.) from Zhoushan and Hangzhou, were collected from rivers, pools, or irrigation canals in Zhejiang Province, China (Appendix 1). All individuals were cultivated individually under axenic conditions in 700 mL of fresh water with 15 drops of general nutrient solution for plants (Asoon Biotechnology Co., Nibong City, Zhejiang, China). Individuals were maintained under natural light indoors at 22–30°C and 61–84% humidity. In most cases, all individuals were harvested seven days after inoculation, dried, and stored in silica gel. Genomic DNA was isolated from 500 mg of silica gel–dried tissues originally collected in the wild using the modified cetyltrimethylammonium bromide (CTAB) method of Fan et al. (2004).

Using ‘*Spirodela polyrhiza*’ as the search phrase, a total of 401 whole‐genome shotgun sequences were downloaded from the National Center for Biotechnology Information’s GenBank database (http://www.ncbi.nlm.nih.gov/). For each DNA sequence, tandem repeats composed of more than two nucleotides and repeated more than twice were searched for using Microsatellite Repeats Finder (http://biophp.org/minitools/microsatellite_repeats_finder/; Benson, 1999). A total of 60 primer pairs were designed to anneal in the flanking regions of identified repeat sequences using Primer Premier 5 (PREMIER Biosoft International, Palo Alto, California, USA) with the following settings: target PCR products 100–300 bp in length, primers 23 ± 2 bp in length, and all other settings set to their defaults (Clark and Gorley, 2001).

Newly synthesized primer pairs (BGI, Shenzhen City, Guangzhou, China) were tested for PCR amplification using DNA from 11 and 12 *S. polyrhiza* individuals from the Zhoushan and Wenzhou populations, respectively. PCR amplification was performed in a thermal cycler (Bio‐Rad, Foster City, California, USA) in a 20‐μL mix containing the following components: 50–100 ng of template DNA, 0.8 mM of dNTP mix, 0.3 μM of each primer, 1× PCR buffer (Mg^2+^ free), 1.5 mM Mg^2+^, and 0.4 μL of *Taq* DNA polymerase (Sangon, Shanghai, China). Microsatellite loci were amplified under the following conditions: 5 min of denaturation at 94°C; 35 cycles of 30 s at 94°C, 35 s at 53–58°C, and 40 s at 72°C; and a final extension of 72°C for 3 min. Fifty of 60 loci were successfully amplified in *S. polyrhiza*. Amplified PCR products of seven to eight randomly selected individuals from each population were resolved on 6% polyacrylamide denaturing gel and visualized by silver staining using pUC19 DNA/*Msp*I (*Hpa*II) (MBI Fermentas, Shanghai, China) as the ladder.

From these initial tests, we detected 19 polymorphic loci (Table 1) and 31 monomorphic loci (Appendix 2) in *S. polyrhiza*. Ten of these polymorphic loci were successfully cross‐amplified in *L. perpusilla*; all loci were monomorphic except for Sp36 and Sp42 (Table 2). These 19 polymorphic loci were genotyped for all *S. polyrhiza* individuals. Amplification of each locus was carried out in 10‐μL reactions that included 30 ng of template DNA, 0.8 mM of dNTP mix, 0.3 μM of each primer, 1× PCR buffer (Mg^2+^ free), 1.5 mM Mg^2+^, and 0.2 μL of *Taq* DNA polymerase (Sangon). Forward primers were labeled with one of several fluorescent dyes (5′ HEX, 5′ TAMRA, or 5′ 6‐FAM; BGI). PCR fragments were genotyped using an ABI 3130 automated sequencer (Applied Biosystems, Foster City, California, USA), and fragment lengths were determined using GeneMapper version 4.0 (Applied Biosystems).

**Table 1:** Characterization of 19 polymorphic microsatellite loci isolated from *Spirodela polyrhiza*

Locus	Primer sequences (5′–3′)	Repeat motif	Allele size range (bp)	*T*_a_ (°C)	Fluorescent dye	GenBank accession no.
Sp4	F: TGAATGCAAAGGATAATTGGG	(AGA)_7_	196–205	55	FAM	563851862
	R: GAGGATGTCAGACCTGGAGCT					
Sp6	F: GAACCTTAATATGCGACCAAG	(TC)_10_	262–270	54	HEX	563851912
	R: CAAGAAAGTCAAATACAGCGG					
Sp10	F: CCATCTGTCGTCCTTTTTCCC	(GA)_16_	186–194	55	TAMRA	563851956
	R: CGCCCCATCACTTATTTCGTA					
Sp12	F: CGGTCCCCGTCCAAAGTACTC	(AG)_15_	269–291	55	FAM	563852003
	R: GCAGCCACCCCCCCTAAAATC					
Sp14	F: GTCCATCCTTCTCAGCACAAT	(CT)_14_	239–271	56	FAM	563852051
	R: CCGTACAAGATCTAAGCCTTT					
Sp16	F: GGATCTGTATATGCCCTCTCT	(CT)_8_	256–262	54	FAM	563852089
	R: GCCGCTATCTCAGGTCTTGCT					
Sp25	F: GGCAGAGACAGAAAGATCATC	(CT)_11_	153–251	53	HEX	563851604
	R: CCTAGTTCCCTAGAGCGAGAG					
Sp28	F: GCTTATATACACCGCAAGGGA	(GA)_8_	146–154	53	HEX	563851677
	R: GGAGGGAAAAAGGTTGACGAC					
Sp29	F: TAAATGACAGATGAAAGCCAA	(ATA)_10_	279–297	56	HEX	563851681
	R: ACTCCAACTCCCACAAGAAGG					
Sp30	F: CGCCTATAAGTAACCCCCTAC	(CT)_9_	199–209	54	TAMRA	563851689
	R: ATCATATCTGCTCGAACCATC					
Sp36	F: GCGTCCTATGAATCGGGGAGC	(AT)_10_	210–226	54	HEX	563851845
	R: TCGAGTCAGCGTTGGGGTGTG					
Sp42	F: TGAGATCAGGCTGGAGCAGTG	(GA)_35_	179–199	55	FAM	563852234
	R: GTTTACGTGGGCTACCAAACA					
Sp43	F: GGAAAATCAGCACGGAGACAC	(GA)_9_	210–240	55	FAM	563852257
	R: TTCACATAGGACGAGGTAGCG					
Sp45	F: AGGATATTCCAGGTGCTCATC	(AT)_12_	281–289	56	FAM	563852311
	R: CCTTGTTTCCGTTCAACTTCT					
Sp47	F: TGGGCCTATGGCGATTAGGGG	(GA)_10_	261–298	56	TAMRA	563852415
	R: GCGGCATCCACGGAGAAAATG					
Sp49	F: GGAATCAACCCAAGTATAGAA	(TTA)_6_	181–185	54	FAM	563852505
	R: CATAGCAGAACTTTAGCGATC					
Sp51	F: CTCGCACATCAGTTCACAGGA	(CT)_23_	254–280	56	HEX	563852540
	R: TCAGACATCTGGCGCAGTAGA					
Sp52	F: GTCCTCCCTTTGATTGCTCGTC	(ATC)_6_	264–266	56	TAMRA	563852544
	R: AAGCATCATGGGCTCTTCAGG					
Sp53	F: AGGACGACGACCTCTACTGCC	(AG)_15_	257–298	58	FAM	563852569
	R: TACGAGTTCTGCGGACCATCA					

Note

*T*_a_ = annealing temperature.

**Table 2:** Amplification success of 19 microsatellite primers across *Lemna perpusilla*.^a^

No.	Sp4	Sp6	Sp10	Sp12	Sp14	Sp16	Sp25	Sp28	Sp29	Sp30	Sp36	Sp42	Sp43	Sp45	Sp47	Sp49	Sp51	Sp52	Sp53
1	—	—	—	—	—	—	—	—	—	—	—	—	—	—	—	—	—	—	—
2	—	—	—	—	—	—	—	—	—	—	—	204	285	—	—	—	—	—	—
3	—	—	—	—	—	—	—	—	—	—	—	232	—	—	—	—	—	—	—
4	—	—	—	—	—	—	—	—	—	—	—	—	—	—	—	179	—	—	—
5	—	—	—	—	—	—	—	—	—	—	—	—	—	—	—	—	—	—	—
6	—	—	—	—	—	—	—	—	—	—	—	—	—	—	—	—	—	—	—
7	—	—	—	—	263	—	—	—	—	—	—	—	—	—	—	—	—	—	—
8	—	—	—	—	—	—	—	—	—	—	—	191	—	—	—	—	—	—	—
9	—	—	—	280	—	—	—	—	—	—	—	191	—	—	—	183	—	—	—
10	—	—	—	—	—	—	—	—	—	—	—	—	240	—	—	183	—	—	—
11	—	—	—	—	—	—	227	138	—	—	—	193–195	—	281	—	—	—	—	—
12	—	—	—	—	—	—	—	—	—	—	—	—	—	—	—	—	—	—	—
13	—	—	—	—	—	—	—	—	—	—	—	—	—	—	—	—	—	—	—
14	—	—	—	—	—	256	—	—	—	—	216–228	—	240	—	—	—	—	—	—
15	—	—	—	—	—	—	—	—	—	—	210	—	204	—	—	—	—	—	—
16	—	—	—	—	—	—	—	—	—	—	—	—	—	—	—	—	—	—	—

Note

— = unsuccessful amplification.

a Numbers indicate PCR fragment lengths.

The number of alleles per locus, levels of observed heterozygosity and expected heterozygosity, and Hardy–Weinberg equilibrium were calculated with TFPGA version 1.3 (Miller, 1997). The inbreeding coefficient and linkage disequilibrium between pairs of microsatellites were calculated by FSTAT version 2.9.3 (Goudet, 2002). The number of alleles per locus varied from two to 13, with a total of 108 alleles scored across 68 individuals. The observed and expected levels of heterozygosity ranged from 0 to 0.8750 and 0.0511–0.8669, respectively (Table 3). The inbreeding coefficient ranged from −0.761 to 1.000, and most loci showed significant deviation from Hardy–Weinberg equilibrium (Table 3), all of which probably resulted from the small, isolated, asexual populations of this species. Linkage disequilibrium for each locus pair across both species was not significant after Bonferroni correction, with the exception of Sp10 × Sp52, Sp36 × Sp52, and Sp43 × Sp52.

**Table 3:** Genetic diversity of 19 polymorphic microsatellite loci in *Spirodela polyrhiza* populations.^a^

Locus	Zhoushan population (*N* = 28)				Wenzhou population (*N* = 24)				Hangzhou population (*N* = 16)				Total (*N* = 68)				
	*n*	*A*	*H*_o_	*H*_e_	*n*	*A*	*H*_o_	*H*_e_	*n*	*A*	*H*_o_	*H*_e_	*n*	*A*	*H*_o_	*H*_e_	*F*_IS_
Sp4	25	3	0.0400	0.1167	24	1	0.0000	0.0000	9	1	0.0000	0.0000	58	3	0.0172	0.0511	0.665
Sp6	23	4	0.1739	0.5556	24	4	0.2500^b^	0.3954	9	1	0.0000	0.0000	56	5	0.1786	0.4249	0.560
Sp10	21	3	0.1905	0.1800	24	1	0.0000	0.0000	10	1	0.0000	0.0000	55	3	0.0727	0.0712	−0.056
Sp12	21	5	0.1429^b^	0.5947	24	3	0.5000	0.5505	9	1	0.0000	0.0000	54	6	0.2778	0.5247	0.427
Sp14	22	7	0.6364	0.7664	24	2	0.5417	0.4034	9	4	1.0000	0.6340	55	7	0.6545	0.6309	−0.121
Sp16	22	4	0.6364	0.6712	24	2	0.6250	0.4388	5	2	1.0000	0.5556	51	4	0.6667	0.5686	−0.219
Sp25	27	12	0.4815^b^	0.8819	24	5	0.3750^b^	0.6179	13	4	0.5385^b^	0.7169	64	13	0.4531	0.8669	0.401
Sp28	23	2	0.8696^b^	0.5101	24	2	0.8333^b^	0.4965	9	2	1.0000	0.5294	56	2	0.8750	0.5005	−0.761
Sp29	24	4	0.0417^b^	0.3324	24	2	0.2917	0.3112	9	1	0.0000	0.0000	57	5	0.1404	0.2846	0.492
Sp30	23	3	0.4348	0.3565	24	1	0.0000	0.0000	9	1	0.0000	0.0000	56	3	0.1786	0.1655	−0.221
Sp36	25	5	0.6400	0.7567	24	4	0.9167^b^	0.6055	9	2	1.0000	0.5294	58	5	0.8103	0.6658	−0.236
Sp42	27	8	0.3704^b^	0.7002	24	4	0.3750	0.3839	12	3	0.0000^b^	0.4203	63	10	0.3016	0.5531	0.434
Sp43	24	4	0.2083^b^	0.5417	24	1	0.0000	0.0000	15	4	0.1333^b^	0.5333	63	6	0.1129	0.3778	0.672
Sp45	25	4	0.0000^b^	0.5845	23	3	0.2917	0.3449	9	1	0.0000	0.0000	57	5	0.1207	0.4703	0.702
Sp47	23	9	0.7391	0.8676	24	6	0.8696	0.7952	10	5	0.8000	0.7737	57	11	0.8036	0.8335	0.022
Sp49	25	3	0.4400	0.5167	23	3	0.5833	0.4512	11	3	0.0000^b^	0.4502	59	3	0.4167	0.4835	0.132
Sp51	23	7	0.3478^b^	0.7130	24	4	0.4583	0.4725	9	2	0.1111	0.1111	56	7	0.3571	0.5449	0.313
Sp52	22	2	0.0000^b^	0.1691	24	1	0.0000	0.0000	9	1	0.0000	0.0000	55	2	0.0000	0.0707	1.000
Sp53	23	8	0.7826	0.8754	24	6	0.7917	0.7793	9	4	1.0000	0.7778	56	8	0.8214	0.8386	−0.003

Note

*A* = number of alleles; *F*_IS_ = inbreeding coefficient; *H*_e_ = expected heterozygosity; *H*_o_ = observed heterozygosity; *N* = number of individuals sampled; *n* = number of individuals successfully amplified.

a Voucher and locality information are provided in Appendix 1.

b Significant deviation from Hardy–Weinberg equilibrium expectations (*P* < 0.01).

## CONCLUSIONS

In this study, 19 loci were polymorphic in 68 tested individuals within three populations. The high transferability of these markers will allow for further population genetic studies of *S. polyrhiza* and other related taxa, which will be useful in bioremediation programs.

## APPENDIX 1 Information for the *Spirodela polyrhiza* and *Lemna perpusilla* samples used in this study. Voucher specimens have been deposited at Zhejiang Ocean University, Zhoushan, Zhejiang, China.

**Table:**

Species	Population ID	*N*	Collection locality	Geographic coordinates	Collector	Voucher no.
*Spirodela polyrhiza* (L.) Schleid.	Hangzhou	16	Jianggan, Xiasha, and Xiaoshan districts, Hanzhou, Zhejiang, China	30°19′10″N, 120°21′53″E	Jia‐ying Ma, Jia‐wei Dai	HZ56
*Spirodela polyrhiza*	Wenzhou	24	Ouhai District, Wenzhou, China	27°55′40″N, 120°42′31″E	Jian‐nan Duan, Jiang‐nan He	WZ30
*Spirodela polyrhiza*	Zhoushan	28	Dinghai and Putuo districts, Zhoushan, China	30°02′18″N, 122°17′4″E	Jia‐meng Wu	ZS8
*Lemna perpusilla* Torr.	Hangzhou	10	Xiasha District, Hanzhou, China	30°18′58″N, 120°20′37″E	Jia‐wei Dai	HZ71
*Lemna perpusilla*	Zhoushan	6	Putuo District, Zhoushan, China	30°01′03″N, 122°16′46″E	Meng‐wei Wang	ZS90

Notes

*N* = number of individuals sampled.

## APPENDIX 2 Characterization of 31 monomorphic microsatellite loci isolated from *Spirodela polyrhiza*.

**Table:**

Locus	Primer sequences (5′–3′)	Repeat motif	PCR fragment length (bp)	*T*_a_ (°C)	GenBank accession no.
Sp1	F: GTGAGAGAAGAAAAGTGAAGG	(TAT)_5_(TAG)_7_	238	54	563851465
	R: CAAGATTTGTGGTTAGGAGCA				
Sp2	F: TCCTATTGACTCGTCGTCTTC	(CT)_9_	249	54	563851470
	R: CGGTGTTCCTCGTATTATCTC				
Sp3	F: GGGTTGTATGATCTTCGGGGG	(AG)_10_	299	56	563851495
	R: GTGCTGGGCTTGACGGGGACT				
Sp5	F: CTATCTTGCACTCTCGTGCGT	(AT)_10_	286	51.5	563851908
	R: TCAATCTTGTTCCTTTGTCGT				
Sp7	F: GTCTGACACAAGTCAACGTGG	(CT)_12_	563	55	563851936
	R: GGTAGAGGGGAGTGAAACAAC				
Sp8	F: GGAACCCAAGGTCAACCTCCG	(ATG)_10_	279	53	563851939
	R: AATAAACAAACACAACCAGCC				
Sp11	F: GAGGGAGGATCGTTATCGTAG	(AG)_8_	270	52.5	563851966
	R: GCCTGTTTGTTGATTAGTTTG				
Sp15	F: CTCTCTCCCGTCCCTCTCCTC	(TC)_11_	291	56	563852062
	R: TCTATCCGTCGGTTCATCTCG				
Sp17	F: TCTCGTCCCCATTTTCAAACA	(CT)_11_	157	54.5	563852092
	R: TGACCCAGAAGAATACCCAAC				
Sp18	F: ACGAGCGGTAGCTGGAGAGCT	(GCG)_7_	219	54	563852112
	R: CCGGTGGAAATTACGGTGGCA				
Sp20	F: AGACTGTCGGCTGTTAGGGGA	(TC)_12_	195	52.5	563852181
	R: TTCAGAACCAGAGGATGTAGG				
Sp23	F: TGGCTCTTGTTGCTCTTTTGC	(GA)_25_	258	52.5	563851258
	R: TGACTTATGACCGGTATTTCG				
Sp24	F: TCTCCATGACAGGTTCTCCCA	(GA)_15_	204	54	563851582
	R: CTCTCACAAGCAAACTTCCCC				
Sp27	F: TTTCTTCTTCTCTTTGGTGCT	(AG)_8_	296	52	563851657
	R: TAGGTTAAAATGTTTTGGCTC				
Sp31	F: ATAATGGCGAGCATGTTGGGA	(AG)_12_	276	55	563851701
	R: ATAGAGGCGATGAGGTTGGGG				
Sp34	F: AAATAACTAAAATGCAACGAG	(TC)_8_	293	54	563851303
	R: TGTAGGAGGATACAAGACTGG				
Sp37	F: ATTCTCTCACATCCTCGTCTC	(AG)_13_	197	53	563851849
	R: CATCATCATCGTATGCTCAAC				
Sp39	F: CGAAGGGACGGAAGAAGACGA	(AG)_10_	223	58	563851400
	R: AGAGGGGAGAGGGATAGGGCT				
Sp40	F: CGTCACCCTCCCCACATTCCA	(AG)_9_	287	58	563851389
	R: ACCAACCGTCTCCCGTCCCAT				
Sp41	F: GTTGGGTTCTGGACTGCTCTA	(CT)_12_	291	53	563852205
	R: TGGGCTCGCAAACACTCTAAT				
Sp44	F: CAAGTGCCGCAGCGAGACGAA	(GAA)_9_	249	58	563852305
	R: CGTGCCAGGCTCTGCACAACA				
Sp46	F: TTGGAGTAACTTCCTGGTGGTA	(GA)_8_	228	54	563852316
	R: TCAAGCTCATAATATCCGATGC				
Sp48	F: TTCTGGGAACCCTCTGTAGCA	(CT)_9_	192	51.5	563852448
	R: GGTCAATACTCTGAAGGCAGTT				
Sp50	F: TCATCATGCGGGCTTTTCAGT	(GA)_8_	148	56	563852522
	R: CGCCGCTCCAACATTTTCAGA				
Sp54	F: GGGCTGGGGCCGCAATGAAAA	(GA)_38_	151	58	563852609
	R: AGGTCGCACCGCCACCAGAAGC				
Sp55	F: AAGCAAGCTCGAAGGAAATAC	(CT)_15_	242	51.5	563852614
	R: GCACAAAGATCCAAGGCACAA				
Sp56	F: GAGACCGCTTCCACATTTCCC	(CGG)_7_	192	57	563852634
	R: CATACAGACCATAAGATCGCACC				
Sp57	F: CACCCTGGAGATAACCAAAGC	(CCG)_8_	100	58	563852681
	R: CTGGTGGAGTCTGGAGAAGGA				
Sp58	F: TGCCCATACTTCTGAACAACA	(CT)_16_	146	52	563852756
	R: CTCATCTTCCTGCCCTACCTC				
Sp59	F: CAGATCCGTGTCCTTCTTCCC	(TC)_9_	297	55.5	563852776
	R: TCTCCCTTTCAAACCGTGCTT				
Sp60	F: AATGGAAGAACGGAGACAGGG	(GA)_12_	222	53	563852780
	R: AGAGGGACAAGCATCGTCAGG				

Note

*T*_a_ = annealing temperature.
